# Impact of COVID-19 Home Confinement in Children’s Refractive Errors

**DOI:** 10.3390/ijerph18105347

**Published:** 2021-05-17

**Authors:** Cristina Alvarez-Peregrina, Clara Martinez-Perez, Cesar Villa-Collar, Cristina Andreu-Vázquez, Alicia Ruiz-Pomeda, Miguel Ángel Sánchez-Tena

**Affiliations:** 1 Faculty of Biomedical and Health Sciences, Universidad Europea de Madrid, 28670 Madrid, Spain; villacollarc@gmail.com (C.V.-C.); cristina.andreu@universidadeuropea.es (C.A.-V.); masancheztena@gmail.com (M.Á.S.-T.); 2 Instituto Superior de Educação e Ciências, ISEC LISBOA, 1750-179 Lisboa, Portugal; clara.perez@iseclisboa.pt; 3 Ophthalmology Department, Hospital Universitario de Móstoles, 28935 Madrid, Spain; aliciaruizpomeda@hotmail.com

**Keywords:** home confinement, COVID-19, myopia, lifestyles, children, vision, outdoors activity, SARS-CoV-2, screen-time

## Abstract

Background: Myopia is a public health problem, with estimations that 50% of the world population will be myopic by 2050. Some environmental factors, such as time spent outdoors, doing near work, and using digital devices, influence the development of myopia in children. Home confinement in Spain has increased these risk factors, so this study aims to investigate the impact of home confinement during the COVID-19 outbreak in the vision of school-aged children; Methods: A cross-sectional study in children between 5 and 7 years old that completed a visual screening and a questionnaire about their lifestyles at opticians in Spain in September and October of 2019 and 2020. Statistical analysis to compare lifestyles pre and post confinement, and vision in 2020 versus a similar cohort examined at the same opticians in 2019, was conducted; Results: Spanish children spent less time outdoors and more time doing near work in 2020 than in 2019 (*p* ≤ 0.001). There was a significant decrease of the spherical equivalent (mean ± standard deviation; 0.66 ± 2.03 D in 2019 vs. 0.48 ± 1.81 D in 2020; *p* ≤ 0.001); Conclusions: Lifestyles of Spanish children changed during the home confinement at the beginning of 2020. Together with changes in their lifestyles, spherical equivalents have decreased, which implies higher figures of myopia for children aged between 5 and 7.

## 1. Introduction

High myopia is now widely recognized as a significant public health problem associated with pathological axial elongation and progressive retinochoroid degeneration in the posterior pole that causes significant visual loss [1]. The increase in the prevalence and severity of high myopia has a clinical and socioeconomic impact throughout the world. By 2050, the World Health Organization estimates that half of the world population will be myopic [2].

Myopia has been recently redefined by the International Institute of Myopia as: “a refractive error in which rays of light entering the eye parallel to the optic axis are brought to a focus in front of the retina when ocular accommodation is relaxed. This usually results from the eyeball being too long from front to back but can be caused by an overly curved cornea and/or a lens with increased optical power. It also is called nearsightedness” [3].

Spain, one of the largest countries in the European Union with a population of 47,351,567 has a National Health System (SNS) based on the principles of universality, free access, equity, and fairness of financing [4]. There are two types of qualified and regulated visual care professionals dedicated to visual health in Spain: ophthalmologists and opticians-optometrists. The population in Spain can check their visual systems through the National Health System or go to a private optical store or a private ophthalmologist. Most eye-care medical and surgical treatments in Spain are covered under the Spanish State healthcare system, but there are not aids for the cost of glasses, contact lenses, or other optical visual aids that are usually fitted in optical stores [5].

Data about refractive error prevalence in Spain shows that in 2000 the prevalence of emmetropia, hyperopia, and myopia was 2.5% in children from 3 to 8 years old [6]. The increase in myopia prevalence in Spanish children was reported in a recent study, with rates that came from 17% in 2016 to 19% in 2017 in children aged between 5 and 7. The number of children with high myopia also increased, from 1.7% in 2016 to 3.6% in 2017 [7]. Regarding other countries in Europe, the prevalence of myopia reached 20% in a 0–9-year French cohort [8]. In the Netherlands, rates of myopia reached 2.4% in a cohort of 6-year-old children [9] and 11% in 9-year-old children [10].

In response to the coronavirus disease 2019 (COVID-19) outbreak, on 14 March 2020, the Spanish government decreed a state of alarm with important restrictive measures on mobility [11]. One of the measures to prevent the spread of the infection was home confinement and school closure. After forty-two days of confinement, children under fourteen years of age were able to go out for a walk, play sports or play for an hour from 12–19 h accompanied by an adult and up to a kilometer from their homes, respecting social distancing. In the case of territories with 10,000 inhabitants or less and whose population density was less than 100 inhabitants/km^2^, departures could be made at any time and at a distance of up to 5 km [12]. On May 11, a transition plan towards the new normality started, which was divided into four phases (from phase 0 to phase 3) by which the mobility of citizens was expanded [13,14,15]. Each phase lasted at least two weeks according to the different autonomous communities. From May 22, children were able to go outside without time limitations. In September 2020, face-to-face educational activity was resumed, adopting a series of prevention, hygiene, and health promotion measures against COVID-19 [16].

In Spain, many 5-year-old children attend pre-primary school, although it is not compulsory. However, all 6–7-year-old children attend primary school [17]. COVID-19 changed Spanish educational routines suddenly in March 2020, resulting in decreased physical activity and increased screen time [17].

Wang et al. have pointed out that this prolonged school closure and home confinement during the COVID-19 epidemic could have negative effects on children’s physical and mental health [18].

In addition to genetics [19,20], there is strong evidence that environmental factors [21] such as time spent outdoors [22,23,24] sustained near vision [10,18] and prolonged higher education [25] play a role in the onset of myopia. Evidence suggests that when children are confined to their homes have much longer screen time and do fewer outdoor activities [26]. It has been also demonstrated that an increase in computer use is associated with myopia development [10,27].

Decreased time spent outdoors and increased sustained near work and digital screen time due to the lockdown and quarantine measurements in all the cities of Spain could have visual repercussions for children. During this last year of pandemic outbreak, the study habits of Spanish children have been modified, increasing the use of digital technology and online e-learning.

Considering that Spain has been one of the most-affected countries with hard restrictive measures because of the pandemic situation, it is important to know how the impact of home confinement during the COVID-19 outbreak has influenced the vision of school-aged children in Spain.

## 2. Materials and Methods

This cross-sectional study in children recruited, using the convenience sampling method, from the XX and XXI “scholar campaign in favor of the visual health” that “Fundacion Alain Afflelou” carried out in 2019 and 2020, respectively, during the months of September and October. This research adhered to the tenets of the Declaration of Helsinki and was approved by the ethics investigation committee of Universidad Europea de Madrid (CEI-UE) under the code CIPI/19/102. All participants that were in the age ranged from 5 to 7 and whose parents or legal guardians declared a clear understanding of the study objectives by signing the informed consent were selected to participate in the study.

The campaign consisted of a vision screening in children between 5 and 7 years old in any of the 325 private optical stores that Alain Afflelou has all over Spain. Optometrists that have been previously trained to follow the same procedures carried out the screening.

The visual screening consisted of the following tests:

- Distance Visual Acuity (VA) with the Snellen chart with and without correction.
- Binocular vision evaluation: cover test, ocular motility, Hirschberg test, Worth test, and near point of convergence
- Objective refraction by retinoscopy at ±6 m
- Subjective refraction by the Maximum Plus to Maximum Visual Acuity Technique (MPMVA) at ±6 m
- Accommodative lag using Nott retinoscopy

Besides the visual screening, parents or legal guardians completed a questionnaire about demographic data and children’s lifestyles. The questionnaire, which has been used in all the annual campaigns from Fundación Alain Afflelou since 2016, was divided into several sections and included the following information:

- Demographical data: sex, age, and place of residence
- Medical record: the reason for the visit, ocular and medical history, and family history
- Questions related to his/her lifestyle: time in outdoor activities, in near activities, and screen time. These questions were modified for children examined in 2020. Parents of these children asked the same questions about children’s lifestyles before and during home confinement.

Based on the objective refraction, Spherical Equivalent (SE) was calculated as the value of the sphere plus the half of the cylinder. Data from the right eye were analyzed and compared in this study.

Regarding the classification of the refractive error, hyperopia was considered as SE ≥ +0.5 D; myopia as SE ≤ −0.5 D, and emmetropia for SE values between −0.5 D and +0.5 D [28,29].

To evaluate time outdoors, screen-time and time spent in near activities, the myopia profile developed by Gifford [29] was used as a reference. As such, time outdoors was considered as low if it was under 1.6 h, moderate between 1.6 and 2.7 h, and high if it was over 2.7 h a day. Regarding the time in near vision activities, it was considered as low if it was under 2 h, moderate between 2 and 3 h, and high if it was over 3 h a day. Finally, the percentage of the time in near vision but using digital devices was registered as screen time. This percentage was considered as low if it was under 25% of the time, moderate between 25% and 50%, and high if the children used digital devices more than the 50% of the time that he/she spent doing near activities.

Visual screening results of the 1600 children examined in 2020 were compared to the 4227 examined in the same campaign made during September and October 2019 to detect changes over years. In the campaign of 2019, children were examined under the same protocol but excluding the questions about confinement.

Qualitative variables were expressed as absolute (n) and relative (%) frequencies. The Shapiro–Wilk test was used to evaluate the parametric behavior of the quantitative variables. Mean values ± standard deviation were given for normal distributions; for non-normal distributions, the data were reported as medians with interquartile range. The Student T-test or Mann–Whitney U test (depending on the normality distribution) was applied to analyze differences between SE of children examined in 2019 and 2020. Right eye SE values were rank-transformed and a MANOVA model including year, age, and their interactions were built to explore the impact of the variables on SE. For all children and each age group (5, 6, and 7-years-old), the proportions of myopes, hyperopes, and emmetropes were evaluated. Chi-square tests were used to assess changes in these proportions between 2019 and 2020, for all children and each age group. To evaluate changes in the lifestyle before and after home confinement, proportions (and their 95% confidence intervals, CI) of children who decreased the time spent outdoors, increased time doing near-distance activities, and using electronic devices were calculated. Differences in right eye SE among children who spent a low, moderate, or high time outdoors were explored, both in the pre-confinement and post-confinement. For this purpose, Kruskal–Wallis tests were performed and followed by a Dunnet’s test (post-hoc analysis) to detect between which outdoors-time groups SE was significantly different. Similarly, right eye SE was compared among children spending high, moderate, and little time doing near-distance activities, and among children spending high, moderate, and little time with electronic devices in both pre and post-confinement scenarios. The analyses were performed using Stata IC v.14 (StataCorp, College Station, Texas, TX, USA), and a significance level of 5% was established.

## 3. Results

1600 children between 5 and 7 years old were examined in September and October of 2020 and compared with the 4227 examined during the same months of 2019. Table 1 shows the descriptive analysis of the sample.

**Figure 1:**
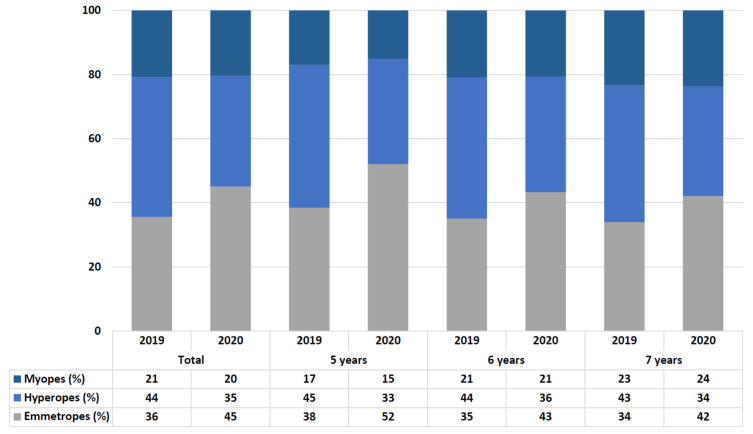
Comparison in the rate of refractive errors in all the children and by ages.

Figure 1 shows the comparison in the percentage of refractive errors in all the samples and by age. The percentage of myopes was the same in 2019 and 2020. However, the percentage of hyperopes decreased and the percentage of emmetropes increased in 2020 vs. 2019, in all the children and analyzing by ages (*p* < 0.001).

**Figure 2:**
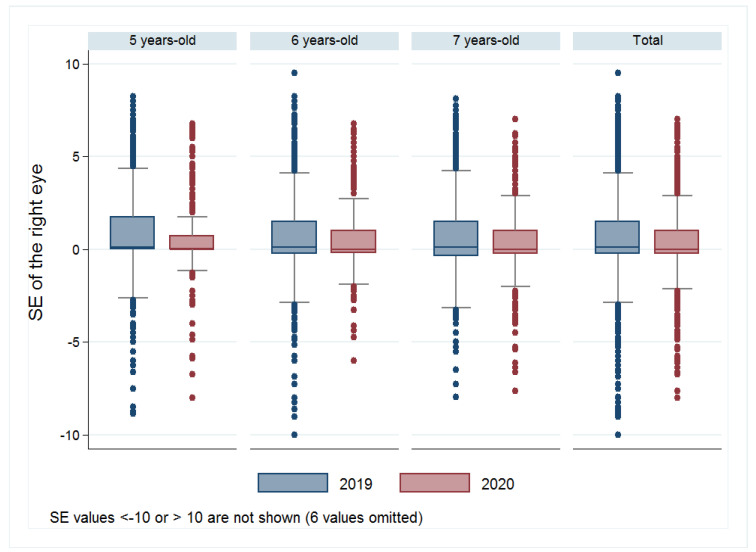
Spherical equivalent in 2019 vs. 2020 by ages and in all the children. The box is determined by Q1 (1st quartile) and Q3 (3rd quartile) and the whiskers are determined by Q3 + 1.5 IQR (upper whisker) and Q1—1.5IQR (lower whisker).

The MANOVA model determined significant differences in SE of the right eye in children examined in different years (F (1,5817) = 17.21; *p* < 0.001) and children from different ages (F (2,5817) = 3.662; *p* = 0.026). Interaction between year and age did not significantly affect SE. Figure 2 shows a significant decrease in the SE of the right eye. The average SE value in 2019 was +0.66 ± 2.03 D, compared to +0.48 ± 1.81 D in 2020 (*p* ≤ 0.001). This decrease was significant in children of 5 years old. The average SE value was +0.77 ± 2.07 D in 2019 vs. +0.56 ± 1.93 D in 2020 (*p* = 0.005). The decrease was just a tendency for 6-year-old children. The average value of SE in 2019 was +0.62 ± 2.07D, while in 2020 it was +0.57 ± 1.73 D (*p* = 0.078). Concerning children aged seven, there was also a decrease in the average of SE. So, the average SE value was +0.61 ± 1.96 D in 2019, while in 2020 it was +0.35 ± 1.82 D (*p* = 0.008). Table 2 summarizes the median, first and third quartile, minimum and maximum values of SE in children evaluated in the years 2019 and 2020 (totals and for each age range).

Regarding lifestyles, 56% of the children changed the amount of time spent outdoors (CI 95%: 53–58), and in the 47% (CI 95%: 45–50) of the cases, this time decreased (*p* < 0.001). Similar data were observed in children of different ages, with changes in 56% (CI 95%: 51–61) and a decrease in the outdoors time for the 46% of the five years old children (CI 95%: 41–51; *p* < 0.001); changes in the 58% (CI 95%: 65–73) and a decrease in the 50% (CI 95%: 55–64; *p* < 0.001) of the six years old children; and changes in the 54% (CI 95%: 50–58) with a decrease in the 46% (CI 95%: 42–50; *p* < 0.001) of the seven years old children.

**Figure 3:**
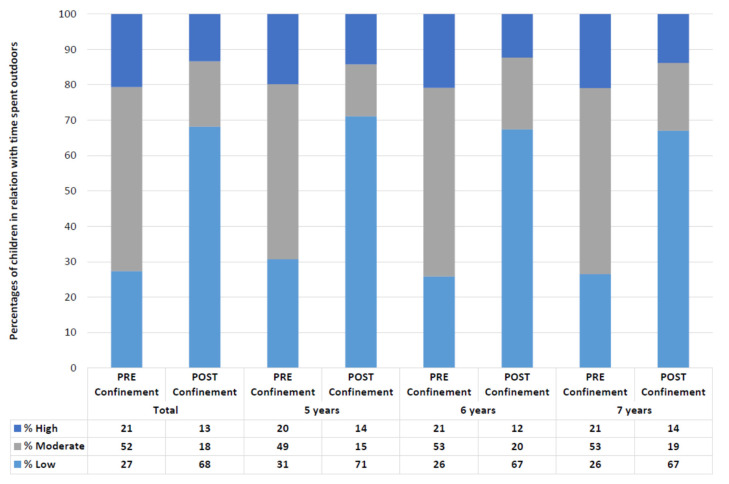
Classification of children depending on the time they spent outdoors in all samples and by ages (Low: <1.6 h a day; Moderate: 1.6–2.7 h a day; High: >2.7 h a day).

Figure 3 shows the percentage of children that spent a low (under 1.6 h a day), moderate (between 1.6 and 2.7 h a day), and high (more than 2.7 h a day) time outdoors before and during home confinement.

Concerning near-vision activities, 49% of children changed the amount of time they spent doing near-distance activities during home confinement (CI 95%: 47–52). 44% (CI 95%: 41–46; *p* < 0.001) of the children increased this time. Similar data were observed in children of different ages, with changes in 51% (CI 95%: 46–56) and increases in the time in near vision activities for 45% of the 5-year-old children (CI 95%: 40–50; *p* < 0.001), changes in 50% (CI 95%: 45–54) and increases in 43% (CI 95%: 39–47; *p* < 0.001) of the 6-year-old children, and changes in 47% (CI 95%: 43–52) and increases in the time doing near vision activities for 43% (CI 95%: 39–47; *p* < 0.001) of the 7-year-old children.

**Figure 4:**
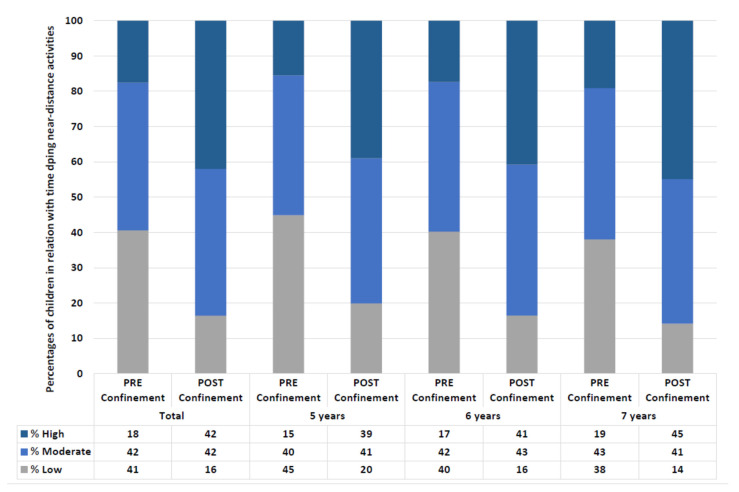
Classification of children depending on the time they spent doing near distance activities in all the sample and by ages (Low: <2 h a day; Moderate: 2–3 h a day; High: >3 h a day).

Figure 4 shows the percentage of children than spent low (less than two hours a day), moderate (between two and three hours a day), and high time (more than three hours a day) doing activities in near vision before and during home confinement.

Changes in the rate of time children use electronic devices while they do near work are also found. 42% of the children changed the amount of time they spent with electronic devices during home confinement (CI 95%: 40–45). 39% (CI 95%: 37–42; *p* < 0.001) of the children increased this time. Similar data were observed for children of 5 years old, with a change in 43% (CI 95%: 38–48) and increases in the screen time for 39% (CI 95%: 34–44; *p* < 0.001). Children of 6 years old followed the same pattern, with a change in 45% (CI 95%: 41–49) of the children, and increases in the screen time for 42% (CI 95%: 37–46; *p* < 0.001). In the case of 7-year-old children, 40% of the children changed the time spent with digital devices (CI 95%: 36–44), and 37% (CI 95%: 33–41; *p* < 0.001) of the 7-year-old children increased this percentage of the near time with digital devices.

**Figure 5:**
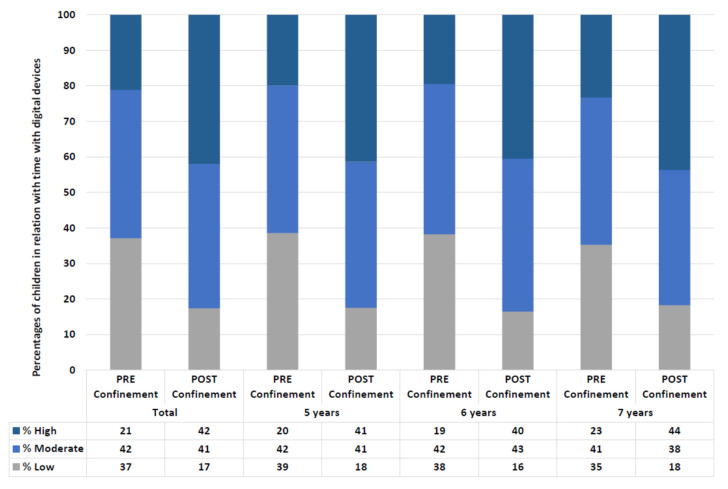
Classification of children depending on the percentage of screen-time while doing near distance activities in all the sample and by ages (Low: <25% of the time doing near activities with digital devices; Moderate: 25–50% of the time doing near activities with digital devices; High: >50% of the time doing near activities with digital devices).

Figure 5 shows the percentage of children that spent a low (less than 25% of the near-work time), moderate (between 25% and 50% of the near-work time), and high (more than 50% of the near-work time) time doing near activities with digital devices.

**Figure 6:**
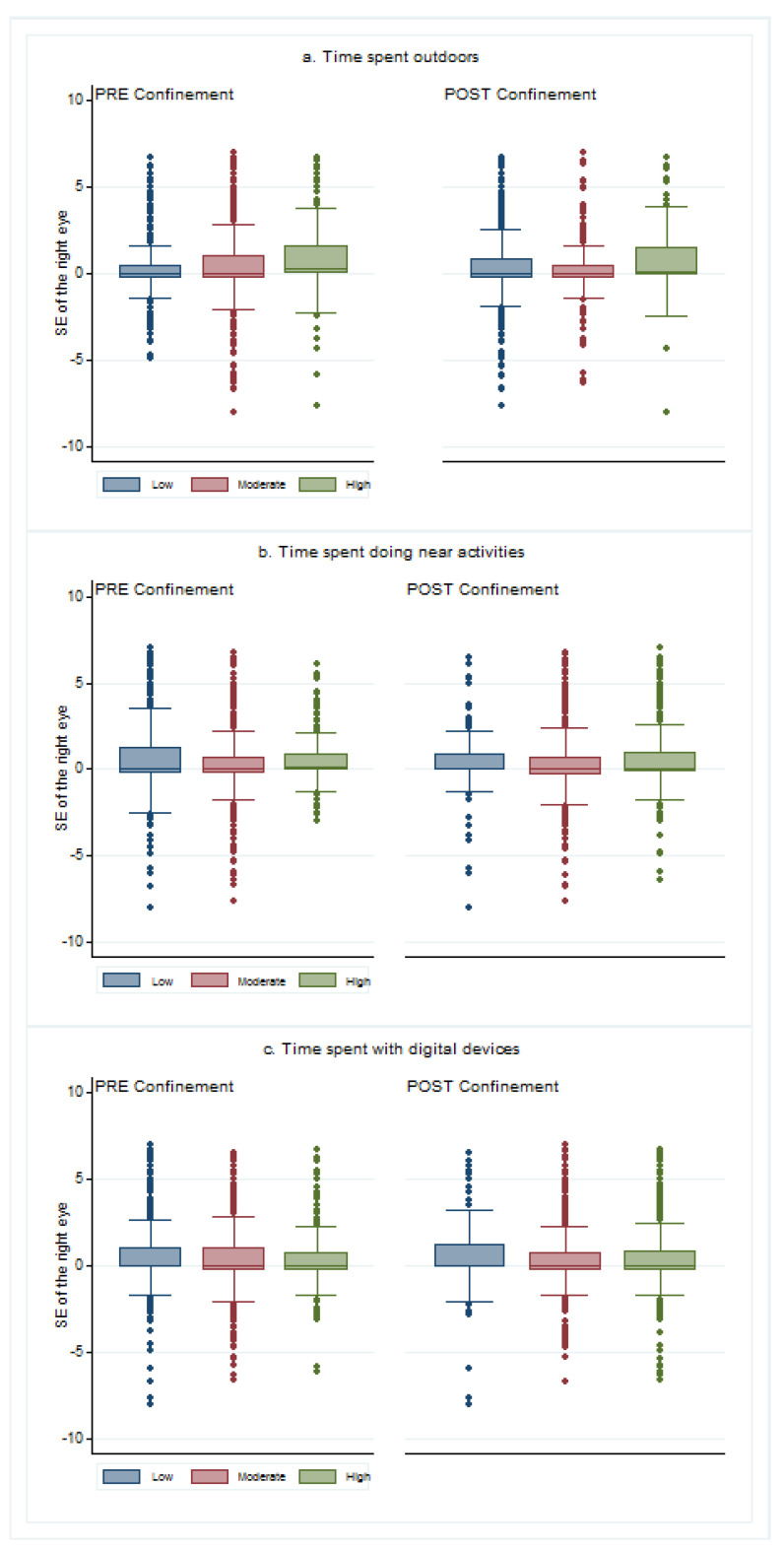
Spherical equivalent of children depending on their lifestyles before and after confinement: (**a**) depending on the time spent outdoors classified as low (under 1.6 h a day), moderate (between 1.6 and 2.7 h a day), and high (more than 2.7 h a day) times; (**b**) depending on the time spent doing near activities, divided into low (less than two hours a day), moderate (between two and three hours a day), and high (more than three hours a day) times; (**c**) depending on the time with digital devices classified as low (less than 25% of the near-work time), moderate (between 25% and 50% of the near-work time), and high (more than 50% of the near-work time) times. The box was determined by Q1 (1st quartile) and Q3 (3rd quartile) and the whiskers were determined by Q3 + 1.5 IQR (upper whisker) and Q1—1.5 IQR (lower whisker).

The relationship between lifestyles and refractive errors showed that children who spent more time outdoors had higher SE in both cases: pre and post confinement (*p* < 0.001 and *p* = 0.049). There was no relationship between the time doing near activities and the SE in either pre (*p* = 0.465) or post confinement (*p* = 0.135). Finally, children that spent more time with digital devices before confinement had lower values of SE (*p* < 0.001), but there were no differences in post confinement (*p* = 0.342). Figure 6 shows the SE values depending on the lifestyles before and after confinement.

## 4. Discussion

The present study is the first research conducted on the consequences that home confinement due to the COVID-19 pandemic has had on the vision of Spanish children. The study used a large sample, with 1600 children between 5 and 7 years old, which can be compared with the sample examined with the same protocol in 2019, with more than 4000 children.

In Spain, the COVID-19 pandemic was especially hard at the beginning of 2020, which led to strict home confinement. For six weeks, children were not allowed to leave their houses at all, and the following eight weeks, the time outdoors was limited to one hour a day.

The changes in Spanish children’s lifestyles have affected many aspects of their lives, increasing their sedentary behaviors [30], decreasing physical activity [31], and even producing changes in their mental health [32]. According to our results, children’s vision has been also affected, with a decrease in the hyperopia rates, an increase in the emmetropia rates, and a decrease in the mean value of SE in children examined in 2020 when compared to 2019. Our study also shows how children in Spain have suddenly decreased the time they spent outdoors, increased the time doing near activities and the percentage of near time that they used electronic devices.

There is enough evidence about how children that spend more time outdoors [33] and less time doing near work activities [34] have less risk of developing myopia than those who do the opposite. The same conclusions have been found in previous studies in Spain [7,35]. For this reason, it is important to know if the vision of children that live in countries with severe confinements have been damaged and myopia has increased faster than expected.

It is important to highlight that we expect changes, even without confinement, in the prevalence of the different refractive errors and the SE values, following the predictions made by Holden et al. for 2050 [2]. However, the great change in lifestyles due to the hard confinement that forced children to be at home for 12 weeks has been such that these changes were higher than expected. Thus, in the last study published on Spanish children, the reference average values for the SE without cycloplegia in more than 8000 children were +0.97 ± 2.15 D for children aged at 5, +0.83 ± 2.00 D for 6-years old children, and +0.66 ± 1.98 D for children of 7 years old [35].

Nevertheless, our study has also limitations. Although optometrists performed objective refraction, and the methodology for refraction is the same for the 2020 and 2019 samples, it was non-cycloplegic refraction, and SE might be overestimated [36]. Further, children’s lifestyles were measured through questionnaires completed by their legal guardians. This gave us subjective values about the time they spent outdoors and near-work time. It should be noted that the data collection has been carried out using convenience sampling, so it presents an inability to generalize the results to the population as a whole, less representativeness of a specific population, and a greater probability of bias in the results.

Some studies have been done in other countries that have suffered similar confinements as China. There, Wang et al. have found that home confinement during the COVID-19 pandemic was associated with a substantial myopic shift for younger school-aged children (6–8 years) [37]. Klaver et al. reviewed the publication of Wang and alerted about the need for intelligent lockdowns for the future, highlighting the importance of considering careful planning of indoor activities and not restricting outdoor activities in children [38]. The same alert has been performed by other eye care professionals [39,40]. Comparing results from Wang el al.’s study to ours, we have found fewer changes in the SE values in the ages included in both studies (6 and 7 years old). Therefore, we observed a decrease in the median values of 0.13 D in all ages while they reported a decrease of 0.23 D for 6-year-old children and 0.28 D for children aged seven.

It is important to know if the changes in the children’s lifestyles are going to revert to the ones they had before COVID-19 or are going to change forever. It seems that some changes, such as the increase in the use of digital devices at school or even the implementation of some hours of homeschooling, could remain. For this reason, it is especially important to conduct future studies to compare 2020 data with 2021 data to forecast the impact the theses changes could have in the long term.

## 5. Conclusions

The lifestyles of Spanish children have been changed during the home confinement at the beginning of 2020. These changes in their lifestyles have led to a decrease in the spherical equivalent, which implies higher figures of myopia for children aged between 5 and 7 years old.

It is recommended to make future studies to compare vision in 2020 with the following years.

## Figures and Tables

**Table 1:** Descriptive analysis of the children.

Year	2019	2020	Total
total (*n*)	4227	1600	5827
females (*n*)	2061	808	2869
females (%)	49%	50%	49%
5 years old (*n*)	1166	409	1575
5 years old (%)	28%	26%	27%
6 years old (*n*)	1469	572	2041
6 years old (%)	35%	36%	35%
7 years old (*n*)	1592	615	2207
7 years old (%)	38%	38%	38%
Unknown age (*n*) *	0	4	4
Unknown age (%)	0%	0.25%	0.07%

* Children with unknown age (*n* = 4) are included in the analyses for all children but they are not considered in the sub-analyses by age groups.

**Table 2:** Right eye spherical equivalent (SE) values for children examined in 2019 and 2020.

Age-Year	Median	Q1	Q3	Minimum	Maximum	*p*-Value *
Total						
2019	+0.13 D	−0.25 D	+1.50 D	−16.75 D	+13.75 D	**<0.001**
2020	0.00 D	−0.25 D	+1.00 D	−8.00 D	+7.00 D	
5-years old						
2019	+0.13 D	0.00 D	+1.75 D	−11.00 D	+13.75 D	**0.005**
2020	0.00 D	0.00 D	+0.75 D	−8.00 D	+6.75 D	
6-years old						
2019	+0.13 D	−0.25 D	+1.50 D	−16.75 D	+9.50 D	0.078
2020	0.00 D	−0.19 D	+1.00 D	−6.00 D	+6.75 D	
7-years old						
2019	+0.13 D	−0.38 D	+1.50 D	−13.63 D	+8.13 D	**0.008**
2020	0.00 D	−0.25 D	+1.00 D	−7.63 D	+7.00 D	

* Mann–Whitney U test was used to compare right eye SE values of children examined in 2019 and 2020. Significant differences (*p* < 0.05) in bold.

## Data Availability

The data presented in this study are available on request from the Alain Afflelou Foundation.
